# Service evaluation of primary care mental health support services in north Wales

**DOI:** 10.1192/bjo.2021.901

**Published:** 2021-06-18

**Authors:** Jawad Raja, Alberto Salmoiraghi, Zeenish Azhar

**Affiliations:** 1 ST5 Betsi Cadwaladr University Health Board; 2 Medical Director Betsi Cadwaladr University Health Board; 3 CT3 Trainee Betsi Cadwaladr University Health Board

## Abstract

**Aims:**

Bringing specialist psychiatrist into PCMHT

Undertaking initial assessments for people Referred by G.P's

Working According to the principle of “Prescribing Interventions”

Decrease number of assessments carried out within secondary Care

**Method:**

County of Wrexham is situated between the lower Dee Valley and the Welsh mountains. It is the largest town in North Wales (140,000)

Since 2013, the total new patient referrals to be seen by Wrexham county consultant psychiatrists has consistently risen

This issue has been dealt with in different ways across North Wales and indeed the whole of Wales

Following a review of services in Wrexham during 2017, it was identified that there was an opportunity to pilot a new model which would allocate a designated Consultant to the local Primary Care Mental Health Team (PCMHT)

The Consultant would work entirely within Part 1 of the Mental Health Measure and would offer specialist opinions to Tier 1 Services

**Result:**

PCMHT team members are maintaining open cases for a significant amount of time rather than the 8–10 sessions that was originally predicted during the implementation of the Mental Health Measures

In order to sustain the service, the minimum number of direct clinical patient contact sessions to be offered by the psychiatrist was up to 4 a week.

During the review period, total number of clinics offered were 51 and a total of 139 patients were offered appointments

Consultants in secondary care covering the same area received exactly 100 less referrals in the first 6 months of the pilot

Main source of referrals to the Tier 1 Consultant came from G.P.'s and the local PCMHT itself

**Conclusion:**

Pilot demonstrated that bringing specialist consultant psychiatrist dedicated to the PCMHT improved the care offered to patients referred by G.P's

Scope of PCMHT needs to extend in order to absorb mild to moderate mental illness and thus avoid patients going into secondary care

This model should be supported, and further resources should be inputted into PCMHT

We should move from a categorical diagnostic referral system to a needs-based intervention where only the most complex cases requiring lengthy interventions shall progress to secondary care

Risk should not be classed as criteria to move patients into secondary care and PCMHT should be able to absorb moderately risky cases

